# The current role and future directions of imaging in failed back surgery syndrome patients: an educational review

**DOI:** 10.1186/s13244-022-01246-z

**Published:** 2022-07-15

**Authors:** Richard L. Witkam, Constantinus F. Buckens, Johan W. M. van Goethem, Kris C. P. Vissers, Dylan J. H. A. Henssen

**Affiliations:** 1 Department of Anaesthesiology, Pain and Palliative Medicine, Radboud University Medical Center, Geert Grooteplein Zuid 10, 6525 GA Nijmegen, The Netherlands; 2 Department of Neurosurgery, Radboud University Medical Center, Nijmegen, The Netherlands; 3 Department of Medical Imaging, Radboud University Medical Center, Nijmegen, The Netherlands; 4 Department of Medical and Molecular Imaging, General Hospital Nikolaas, Sint-Niklaas, Belgium

**Keywords:** Failed back surgery syndrome, Signs and symptoms, Diagnostic imaging, Magnetic resonance imaging, Artificial intelligence

## Abstract

**Background:**

Failed back surgery syndrome (FBSS) is an umbrella term referring to painful sensations experienced by patients after spinal surgery, mostly of neuropathic nature. Adequate treatment of FBSS is challenging, as its etiology is believed to be multifactorial and still not fully clarified. Accurate identification of the source of pain is difficult but pivotal to establish the most appropriate treatment strategy. Although the clinical utility of imaging in FBSS patients is still contentious, objective parameters are highly warranted to map different phenotypes of FBSS and tailor each subsequent therapy.

**Main body:**

Since technological developments have weakened the applicability of prior research, this educational review outlined the recent evidence (i.e., from January 2005 onwards) after a systematic literature search. The state of the art on multiple imaging modalities in FBSS patients was reviewed. Future directions related to functional MRI and the development of imaging biomarkers have also been discussed.

**Conclusion:**

Besides the fact that more imaging studies correlated with symptomatology in the postoperative setting are warranted, the current educational review outlined that contrast-enhanced MRI and MR neurography have been suggested as valuable imaging protocols to assess alterations in the spine of FBSS patients. The use of imaging biomarkers to study correlations between imaging features and symptomatology might hold future potential; however, more research is required before any promising hypotheses can be drawn.

## Key points

- The correlation between morphological changes after spinal surgery and the patient’s symptoms is still poorly understood, and more research is needed to clarify this relationship.
- With the options of modern imaging, especially the metal artefact reduction techniques, the morphology of the postoperative spine can be displayed in detail.
- Contrast-enhanced MRI and MR neurography have been suggested to favor conventional, non-contrast-enhanced MR imaging protocols in order to detect changes in the spine of FBSS patients in small study samples.
- Preoperative radiography might hold predictive utility for the development of FBSS, thus guiding a potential prehabilitation program.
- The search for imaging biomarkers which correlate with symptoms of FBSS forms a largely understudied field of research, and robust prospective trials are warranted.

## Clinical challenge of failed back surgery syndrome

Failed back surgery syndrome (FBSS), also known by the recently proposed terms such as chronic pain after spinal surgery [1] and persistent spinal pain syndrome [2], is considered one of the iatrogenic etiologies of chronic low back pain (LBP). FBSS is defined as persistent lumbar pain despite surgical intervention or recurring pain emerging after spinal surgery, mostly of neuropathic nature [3]. Compared to other chronic pain syndromes, FBSS patients report significantly lower quality of life scores [4], higher levels of pain and disability, and are more often unemployed [5]. FBSS is known to be difficult to treat as its underlying etiology is multifactorial and still not fully elucidated [6, 7]. With regard to diagnostic imaging, FBSS imaging is complicated and relatively understudied, resulting in the absence of an evidence-based imaging strategy. Treatments for FBSS can be generally categorized into physical therapies, rehabilitation programs, psychological and educational sessions, pharmacological agents, interventional procedures (e.g., adhesiolysis), intrathecal drug delivery, redo surgery, and neuromodulation [7, 8]. In general, a curative therapy is rarely available for a patient suffering from FBSS.

When neither conservative therapies nor another surgical intervention are likely to relieve pain sufficiently, neuromodulation treatments such as spinal cord stimulation (SCS) could be provided as a symptomatic treatment. The results of SCS are promising in enhancing pain relief, quality of life, and functional capacity [9, 10]. Specifically in FBSS patients, SCS therapy has been reported as more efficacious than redo spinal surgery [11], which may be declared by the high risk of failure concerning redo surgery in FBSS patients, as the insufficiently addressed initial pathology might become accompanied by novel sources of pain [12].

Here, we must untangle the gordian-knot situation of diagnostic imaging in FBSS. On the one hand, as the role of diagnostic imaging in FBSS has not been investigated thoroughly, no harmonized guidance on its use has been proposed. On the other hand, the lack of standardization of imaging strategies severely hampers the definition of uniform imaging guidelines in FBSS patients. Also, the lack of robust diagnostics impedes the more objective identification of FBSS patients suitable for clinical trials and thus hinders the development of thorough management guidelines since accurate identification of the source of pain remains impossible.

**Fig. 1:**
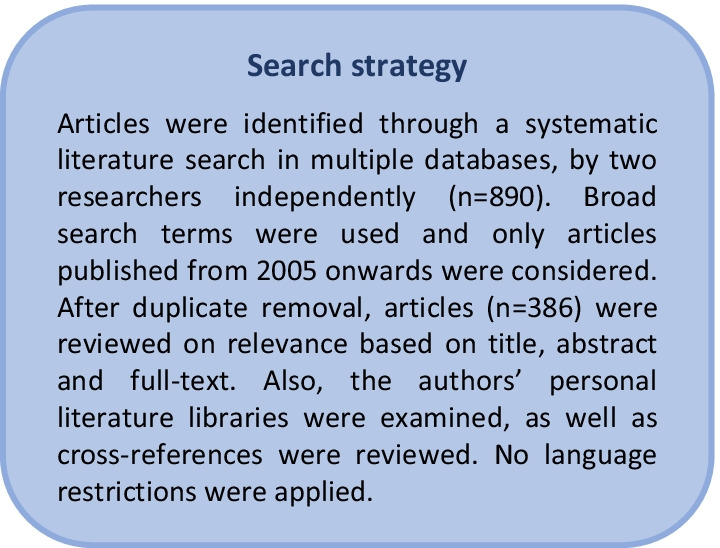
Search strategy. Overall, we noticed that many studies did not provide a conclusive statement on what patients were studied or what symptoms the studied patients suffered from. Consequently, these studies were excluded as we were unsure whether FBSS patients were studied. Also, some studies focused on the preoperative setting, and only a few studies explored any possible correlations between imaging features and symptomatology. Since the reviewed papers in the current study were nonrandomized and mostly explorative, no quality assessment tools were available. Therefore, the level of evidence and risk of bias could not formally be assessed. The lack of randomized or diagnostic accuracy trials could be declared by the current obscurity of what imaging features may clarify FBSS symptomatology

On spinal imaging, (neuro)anatomical correlates which can be observed in FBSS patients comprise spinal stenosis (central, lateral or foraminal), disk herniation (new, residual or recurrent), degenerative changes (disk or facet degeneration, especially when accompanied by inflammatory pathologies), joint pathologies (e.g., facet syndrome), inflammatory changes (e.g., arachnoiditis, spondylodiscitis or radiculitis), nonunion, misplaced or broken material or subsidence, sagittal or coronal imbalance, and signs of nerve injury. However, no anatomical correlates were observed in some patients [7, 13–15]. Therefore, exploration of the patient’s history, physical examination and diagnostic analgesic injections remain crucial alongside imaging [6]. As a conclusive relationship between FBSS symptomatology and imaging features remains unknown, the clinical significance of imaging in the postoperative lumbar spine is still questionable, especially concerning the detection of supportive findings which confirm the diagnosis of FBSS. Hence, there is an urgent need for objective parameters (e.g., imaging biomarkers) in order to identify different phenotypes of FBSS and tailor subsequent treatments for these patients, thereby improving outcomes. The purpose of this educational review is to provide an overview of diagnostic imaging techniques in FBSS patients, including developing imaging possibilities which might hold future potential, such as functional MRI (fMRI), nuclear neuroimaging strategies and Artificial Intelligence (AI)-based tools. The recent literature was outlined as the technological developments have weakened the applicability of prior research (Fig. 1).

## Radiography of the spine in FBSS

Plain radiography, sometimes including dynamic imaging, is commonly performed as the first imaging diagnostic. Besides clarifying degenerative changes, it has the unique ability to evaluate sagittal and coronal alignment in standing position and may therefore diagnose functional spondylolisthesis in the presence of normal findings on magnetic resonance imaging (MRI) [16]. Regarding spinal instability, weight-bearing computed tomography (CT) might be superior to dynamic radiography, even in cases with artefacts caused by metallic instrumentation [17]. Also, radiography could evaluate the positioning and mechanical condition of implants, their osseointegration and fusion status. Limitations of radiographic imaging are well known and include poor soft-tissue contrast and two-dimensional nature.

Dhagat et al. (2017) [18] disclosed that radiography is of limited value in determining the source of FBSS symptomatology, mainly due to its limited ability to assess the soft tissues and disks. The authors did not elaborate on clarifications for the limited value of radiography, nor in correlation to MRI scanning, despite that an MRI scan was also obtained in all patients. Radiography has also been investigated to evaluate whether it could be utilized as a predictive tool for the development of FBSS. A long-term follow-up study (> 5 years) by Xia et al. (2008) [19] evaluated pre- and postoperative radiography in a recovery and non-recovery group (i.e., FBSS patients who suffered from residual LBP) after single-level laminectomy to treat lumbar spinal canal stenosis. They concluded that the preoperative lumbar lordosis angle and lumbar range of motion are significant predictors for the development of residual LBP, indicating that patients with flatback syndrome or decreased lumbar spine mobility are prone to develop FBSS. Contrarily, the percentage of slip, intervertebral rotation angle and preoperative scoliosis or slippage were not significantly correlated. Though, the used cutoff values for allocating patients into a certain group were not specified.

**Fig. 2:**
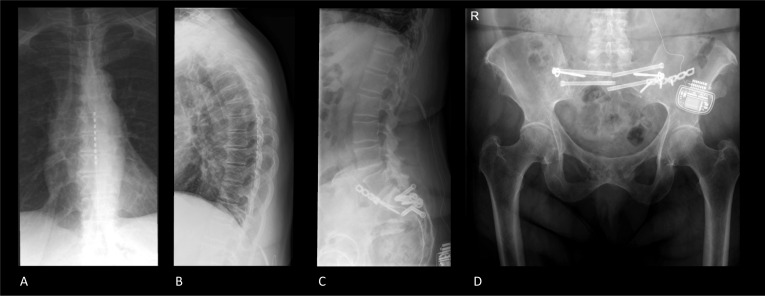
Radiographs of the thoracic- and lumbar spine and pelvic region of an FBSS patient. **A** The spinal cord stimulation electrode projects centrally at the level of Th8–Th10 on the anterior–posterior view. **B** The lateral view shows that the electrode projects over the posterior one-third of the spinal canal at levels Th8–Th10. **C**, **D** The internalized pulse generator can be observed projecting over the left gluteal region. Extension cables can be observed between the internalized pulse generator and the spinal cord stimulation electrode. Osteosynthesis materials can be observed projecting over the sacroiliac joint bilaterally and the lumbosacral joint. The vertebral column shows no anterior or posterior displacement. Degenerative changes in the thoracic and lumbar vertebrae can be observed with anterior and lateral osteophytes

Specific radiography imaging protocols for patients suffering from FBSS have not been described in the literature. After neuromodulation treatment of FBSS, however, the electrode of the SCS system can be appreciated projecting over the spinal column on radiography. On a lateral radiograph, the electrode of the neuromodulation system will project in the posterior third of the spinal canal as the electrode elicits dorsal column stimulation. Despite that patients suffering from FBSS complain of low back pain, the neuromodulation electrode is placed extradurally over the lower thoracic spine, above the level of the conus medullaris (Fig. 2A, B). The electrode is connected with an internalized pulse generator which is placed in the lower abdomen or gluteal region. The internalized pulse generator can sometimes be observed on radiographs of the lumbar spine or the pelvic region (Fig. 2C, D).

## CT imaging of the spine in FBSS

During the early 1980s, CT scanning resulted in a conclusive diagnosis in less than 5% of the FBSS patients [20]. However, two studies published in 2002 showed that the pathology underlying FBSS could be clarified in over 90% of the cases when using a combination of imaging techniques and other diagnostics [14, 15]. Regarding FBSS patients with predominant leg pain, foraminal stenosis and nonunion were most efficiently diagnosed by CT scanning [14].

**Fig. 3:**
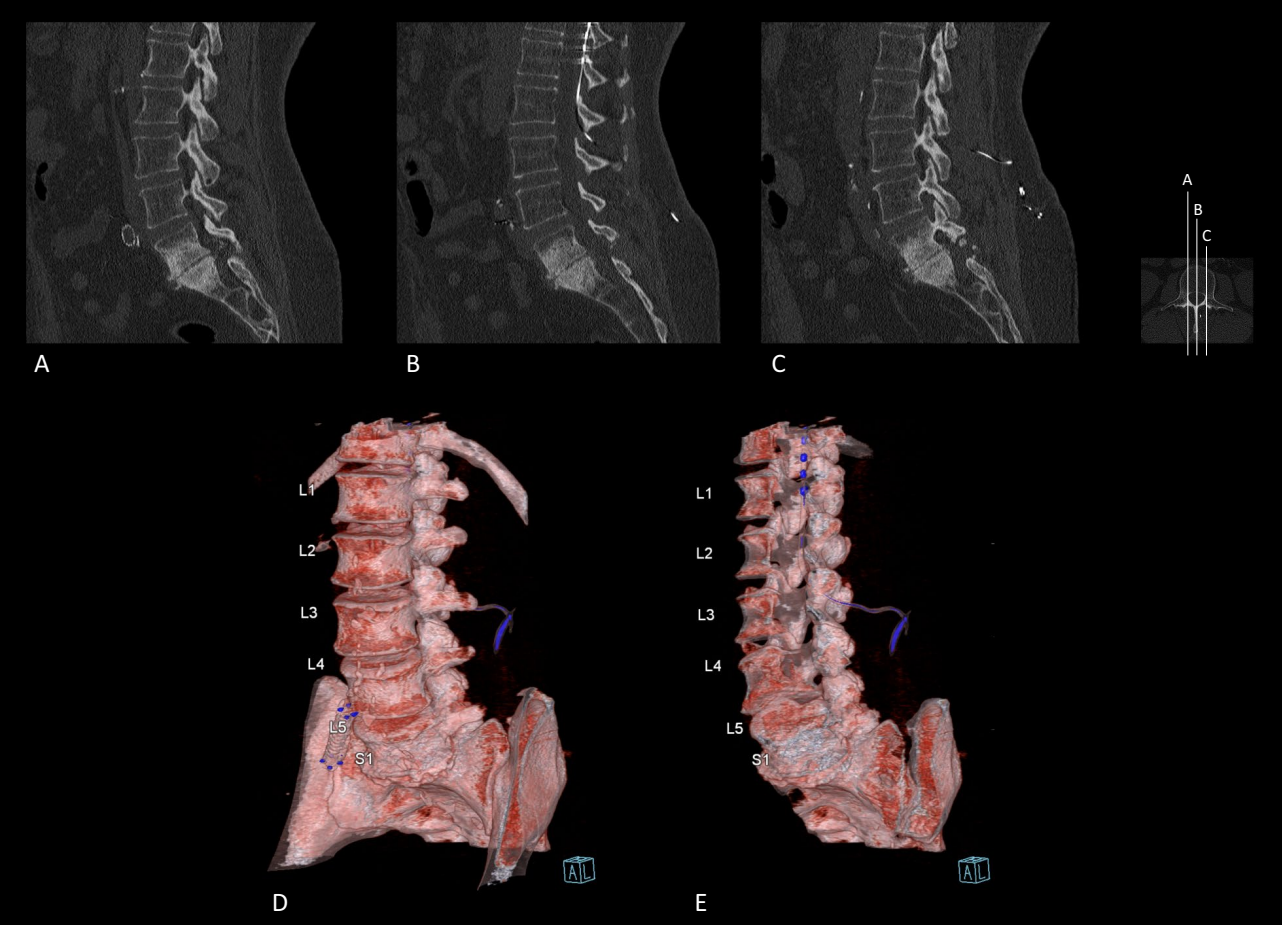
Lumbar spine CT images in an FBSS patient with an implanted spinal cord stimulation system. **A**–**C** Sagittal reconstructions through the vertebral column as depicted by the axial miniature on the right-hand side. The spinal cord stimulation electrode can be observed lining the ventral aspect of the dorsal lamina on level Th12–L1. Sclerosis of L5 and S1 can be observed. Additionally, at level L5–S1, bilateral laminectomy has been carried out. **D** Three-dimensional reconstructions of the spine of the same FBSS patient. The connection of the extension cables to the internalized pulse generator can be appreciated in blue at level L3–L4. **E** Virtually dissected three-dimensional model of the spine of the same FBSS patient. The spinal cord stimulation electrode can be appreciated as multiple blue dots in the spinal canal. The extension cable can be followed into the paraspinal space

**Fig. 4:**
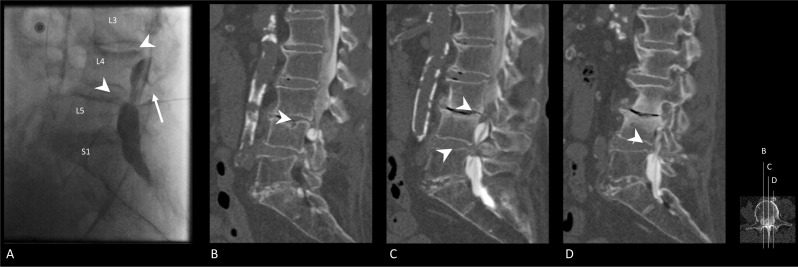
Fluoroscopic myelography and CT myelography images after lumbar punction and intradural injection with iodine-contrast medium. **A** Fluoroscopic myelography image after lumbar punction. The lumbar punction needle is annotated by the white arrow. Iodine-contrast is inserted into intradural space and shows spinal stenosis (white arrowheads) at level L4–L5 and, to a lesser extent, at level L3–L4. **B**–**D** Sagittal reconstructions of CT myelography (localization as depicted by the axial miniature on the right-hand side). The injected iodine-contrast is observed as radiopaque fluid in the dural sac. These images confirmed the observed spinal stenosis at level L4–L5 and L3–L4 as observed on fluoroscopic myelography. Spinal stenoses are annotated by use of the white arrowheads

CT is still the preferred imaging modality when evaluating bone structures, osseous alterations (e.g., nonunion or osteosynthesis complications) or the condition or positioning of implants [21]. For example, a misaligned implant (i.e., displaced interpedicular metallic screw) was confirmed by non-contrast CT, while the MRI findings were inconclusive due to imaging artefacts caused by the metallic implant [18]. Furthermore, CT is indicated when MR imaging is contra-indicated, such as when a patient is implanted with an unconditional MRI device such as an SCS system (Fig. 3). In such patients, CT-Myelography can be a valuable imaging modality to diagnose compression of the thecal sac or nerve roots by a disk herniation or spinal stenosis (Fig. 4). Contrarily to titanium alloy implants, artifacts resulting from ferromagnetic alloy instrumentation could impede data assessment, which might be overcome by performing CT-Myelography [22], though state-of-the-art metal artifact reduction (MAR) techniques help enhance the visualization of key anatomical structures in patients with instrumented spinal fusion. Utilizing an iterative MAR, soft tissues became better visualized, and the length of linear artifacts dropped significantly [23]. Another study showed that dual-energy CT, in comparison with single-energy CT, reduces artifacts and improves image quality in instrumented cervical, thoracic and lumbar spines [24]. The most optimal MAR approach should be selected for each type of instrumentation or surgical procedure in order to further optimize the diagnostic value of CT in the postoperative spine.

## SPECT/CT imaging of the spine in FBSS

The combination of single photon emission computed tomography (SPECT) and CT provides both an anatomical view as a functional pain source evaluation in search of a site with abnormal metabolic activity. These assets are superior to bone scans with planar views, as they fail to accurately distinguish between increased bone turnover rate in either the anterior or posterior sites of the vertebrae. Moreover, the 3D aspect of SPECT/CT has shown to be an accurate localizer of bone turnover at the disk and pars interarticularis [25] and helps in detecting facet joint and disk pathology in chronic LBP patients [26].

**Fig. 5:**
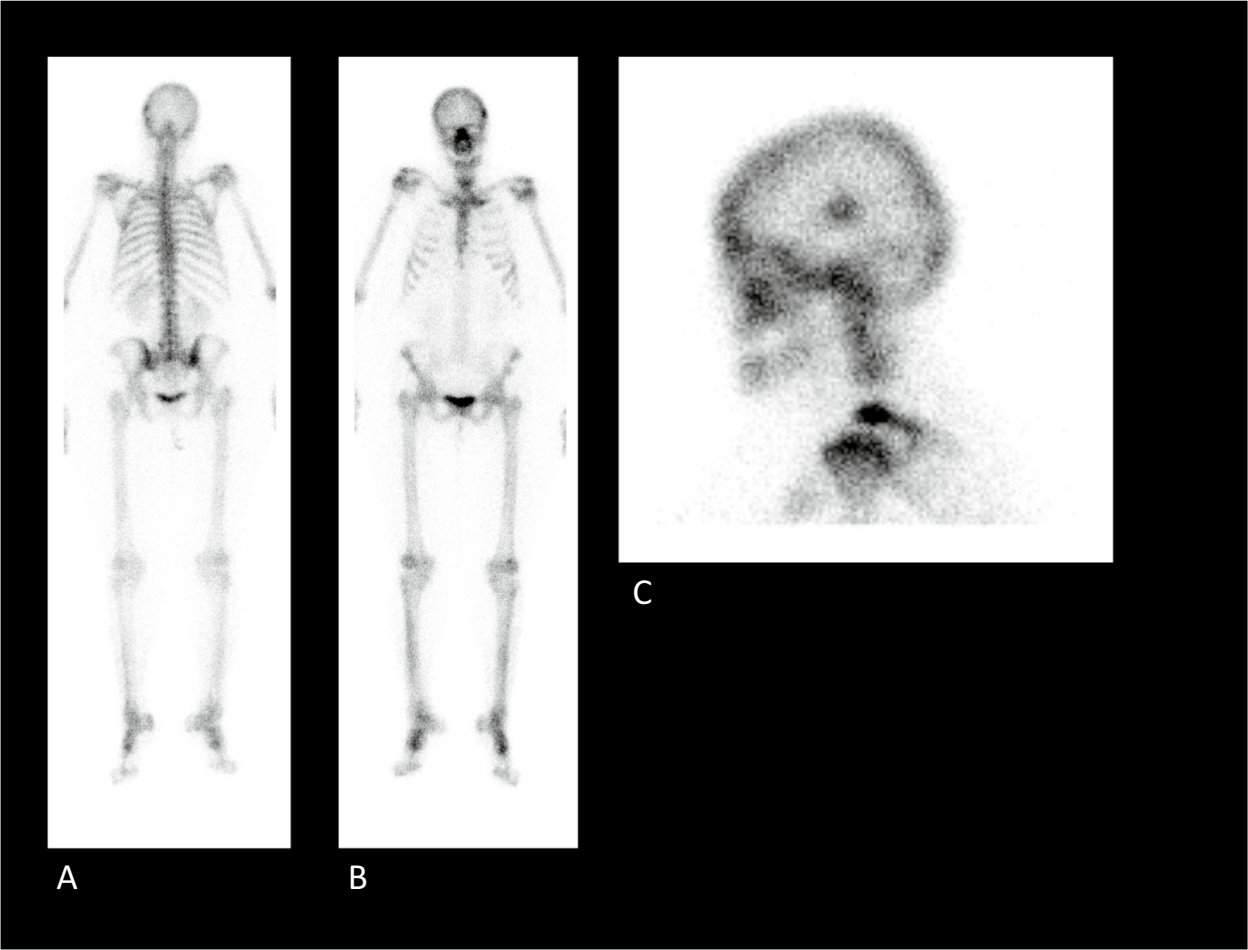
Planar skeletal scintigraphy of an FBSS patient using technetium-99m hydroxydiphosphonate (Tc-99m HDP) to investigate rheumatological disease in the tarsal and metatarsal joints. **A** Posterior full-body planar image; **B** anterior full-body planar image; **C** Lateral skull planar image. Although Tc-99m HDP skeletal scintigraphy was carried out to assess the tarsal and metatarsal joints, full-body planar images were also acquired. In corroboration with the findings of Huang et al. [29], planar images show no specific features of FBSS. Findings were consistent with (nonspecific) degenerative changes in the spinal column and the bilateral tarsometatarsal joints. Focal round accumulation of tracer was observed in near the left parietal bone. This incidental finding was consistent with left parietal meningioma

**Fig. 6:**
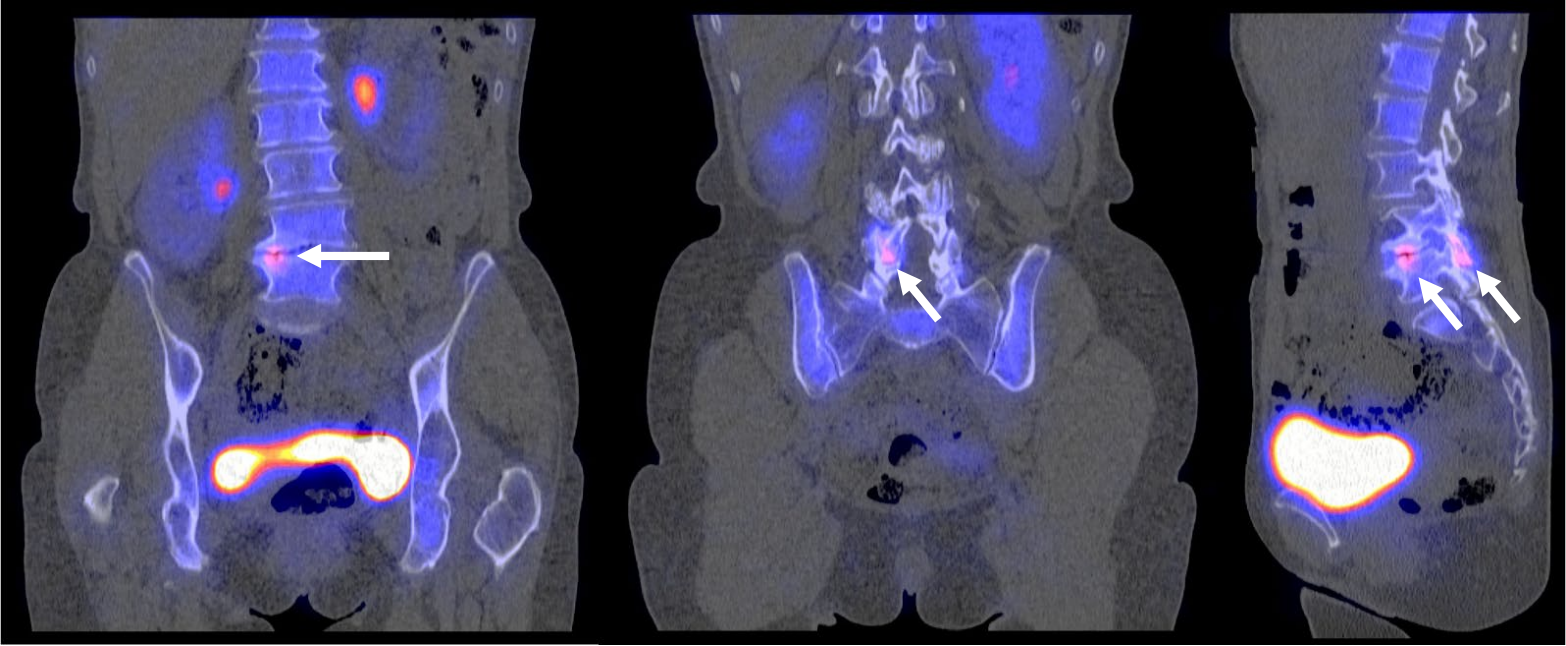
SPECT/CT of an FBSS patient using Tc-99m HDP to investigate the cause of persistent pain after left-sided laminectomy at level L5–S1. Focal radiotracer accumulation was observed at level L4–L5, both near the intervertebral disk and the right zygapophyseal joint. Tracer accumulation was considered to reflect active osteoarthritic changes. No reoperation was carried out

When conservative treatments and analgesic injections fail to relieve pain sufficiently, reoperation is often considered to treat FBSS, especially instrumented spinal fusion [27]. As the success rate of reoperation decreases after each additional surgical intervention [28], correct identification of the source of pain is important since it allows physicians to determine whether reoperation is indicated and to plan any subsequent surgery adequately. One study reported correlations between SPECT/CT findings and revision surgery outcomes [29]. FBSS patients with pathological findings on either radiography or MRI were enrolled in the surgical group (*n* = 7), while the others were enrolled in the medical group (*n* = 9). Within the surgical group, three patients showed positive SPECT/CT findings (*i.e.,*, facet arthritis, disk inflammation or pedicle screw loosening). It was noted that these patients responded better to reoperation than the remaining four patients who showed negative SPECT/CT findings. The four remaining patients were found to have a similar outcome compared with the patients allocated in the medical group. The authors concluded that SPECT/CT could be beneficial in elucidating the source of pain, particularly in patients suspected to suffer from an inflammatory back pathology. Their results should be interpreted carefully due to a low sample size, and we reckon that MRI should also be performed to confirm inflammatory aspects such as edema. No hypotheses declarative for the positive correlation between the abnormal SPECT/CT findings and surgery outcome were given. Nevertheless, these results encourage the further investigation of the role of SPECT/CT imaging in selecting FBSS patients for revisionary fusion surgery (Figs. 5, 6). However, no other findings on this specific topic were found. Also, at our own institution, correlations between SPECT/CT imaging findings and postoperative outcomes remain elusive.

## Conventional MRI of the spine in FBSS

**Fig. 7:**
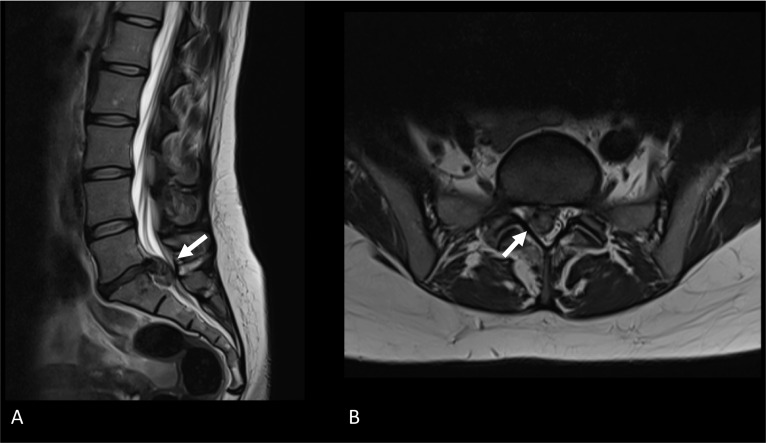
MRI of the lumbar spine demonstrates a large disk herniation on level L5–S1. **A** Sagittal T2-weighted MRI depicts disk protrusion at level L5–S1. A close relation with the cauda equina roots can be observed. **B** Axial T2-weighted MRI shows the protruding disk at level L5–S1 occupying part of the central zone and right subarticular zone. Cauda equina roots are displaced to the contralateral side of the dural sac

**Fig. 8:**
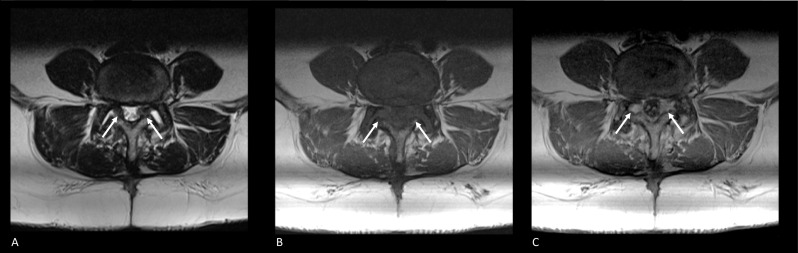
Axial MRI of the postoperative lumbar spine at level L4–L5. **A** Axial T2-weighted image showing soft tissue occupying the subarticular zone bilaterally. Tissue shows to have an intermediate signal intensity. Cauda equina roots are displaced and course more central in the dural sac. **B** Axial T1-weighted image showing the tissue characteristics of the known soft tissue in the bilateral subarticular zone. On native T1-weighted images, an intermediate signal intensity is observed. **C** Post-contrast axial T1-weighted image showing homogeneous enhancement of the known soft tissue in the subarticular zone bilaterally. Based on this enhancement, a suggestion of postoperative epidural fibrosis was made

MRI offers superior soft-tissue contrast than radiography and CT imaging, making it more proficient in assessing neural tissues and detecting inflammatory or lipomatous osseous alterations. Obtaining multiplanar images is easier with MR imaging, but patients have to lie still for a relatively long time. Especially for chronic pain patients who also endure pain in rest, remaining completely idle could be a hard task. Another contra-indication for MR imaging is the presence of MRI unconditional implants. Although some implants can be turned off during scanning, their software and settings could still be disturbed, leading to a malfunctioning device afterward. Another hindrance to performing an MRI scan might be metallic implants, which could lead to imaging artefacts. However, numerous MAR sequences are available to diminish the extent and intensity of susceptibility artefacts, which are MRI artefacts caused by magnetic field distortions. These sequences generally address either ‘in-plane’ or ‘through-plane’ artefacts. The manner results from metal presence in the image plane, while the latter concerns distortion from an adjacent plane. The principles behind such sequences, together with multiple examples, were outlined by Hargreaves and colleagues [30]. MAR sequences to address both ‘in-plane’ and ‘through-plane’ artefacts are also available, such as ‘multi-acquisition variable resonance image combination’ [31] and ‘slice-encoding for metal artefact correction’ [32]. Selecting the most suitable MAR sequence in the postoperative patient is pivotal for adequate radiological evaluation and treatment planning. Lastly, although rare, administering contrast agents could lead to adverse events. However, seen from a risk–benefit perspective in FBSS patients without previous reactions to contrast, physicians should not withdraw from such contrast agents, as contrast-enhanced MRI has become the gold standard for differentiation between epidural fibrosis and residual or recurrent disk herniation [33] (Figs. 7, 8).

Almeida and colleagues (2008) [34] investigated epidural fibrosis as a possible contributor to FBSS-related symptoms using MRI. Their scoring of the degree of fibrosis was based on criteria defined by Ross and colleagues [35]. Starting from the center of the spinal canal, axial T1-weighted images on three levels were split into four quadrants. The degree of fibrosis ‘*F*’ was graded on a scale from 0 to 4 (i.e., grade 0, no fibrosis; grade 1, *F* ≤ 25%; grade 2, < 25% *F* ≤ 50%; grade 3, < 50% *F* ≤ 75%; grade 4, *F* > 75%). Then, the degree of fibrosis was correlated with clinical outcomes (i.e., level of lumbar and leg pain, level of leg pain, McGill Pain Questionnaire and Straight Leg Raising test) and patient characteristics. The authors concluded that epidural fibrosis should not be considered a contributor to FBSS-related symptoms, as no statistically significant correlations to clinical outcomes and patient characteristics were observed at three months follow-up. The developmental process of mature fibrotic tissue is not finished earlier than six months postoperatively [36], implying that correlations to a clinical outcome might alter as the composition of fibrotic tissue will change during this early postoperative period. Nonetheless, their findings coincided with previous literature at more than six months follow-up [37, 38]. Contrarily, at six-month follow-up, a double-blind, randomized controlled trial (1996) showed a positive probability between the amount of fibrosis and recurrent radicular pain as measured by the pain intensity scores denoted by patients as ‘most severe pain.’ However, this probability was not present when the average pain intensity scores were analyzed, and their logistic regression did not reach statistical significance [39]. Overall, imaging findings suspected for epidural fibrosis should be considered the cause of the patient’s symptoms with ultimate caution.

**Fig. 9:**
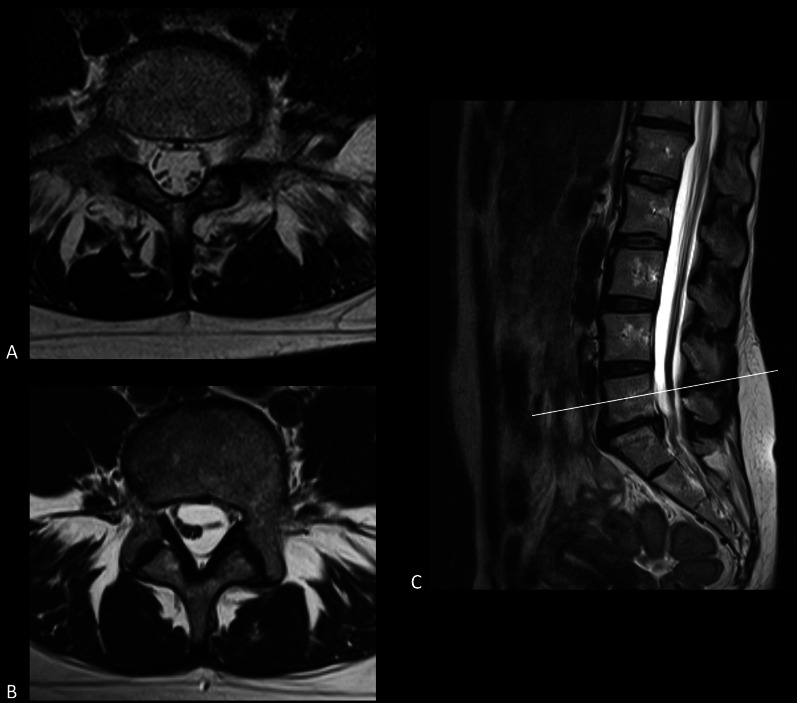
T2-weighted MRI of the same FBBS patient at two-time intervals. **A** First available axial T2-weighted MRI section at the level of the fifth lumbar vertebra. Cauda equina roots are seen to float in ample cerebrospinal fluid. **B** Axial T2-weighted MRI section at the level of the fifth lumbar vertebra of the same patient after spinal intervention at level L5–S1. The patient complains of recurrent right-sided leg pain, sensory changes, and motor weakness. MRI show new clumping of the cauda equina roots on the right side of the dural sac. These features were consistent with arachnoiditis. **C** Mid-sagittal T2-weighted MRI, which also shows the clumping of cauda equina roots

In line with the previous literature [33], Dhagat et al. (2017) concluded that contrast-enhanced MRI is the imaging modality of choice within the FBSS population. They disclosed a 100% success rate in providing an explanation for the patient’s complaints through contrast-enhanced MRI. These explanations included recurrent or residual disk herniation (*n* = 16; 53%), epidural fibrosis (*n* = 6; 20%), coexistence of disk herniation and fibrosis (*n* = 3; 10%), arachnoiditis (*n* = 2; 7%) (Fig. 9), postoperative discitis (*n* = 2; 7%) and malaligned spinal fixation implant (*n* = 1; 3%). Although their 100% success rate of identifying the pathology behind FBSS seemed promising, it must be noted that additional diagnostics confirmed only two diagnoses (6.7%) (i.e., two times postoperative spondylodiscitis confirmed with CT guided cytology aspiration).

Nevertheless, similar explanations for the source of FBSS-related symptoms were presented by Dessouky et al. (2018) [40], who utilized similar MRI equipment but without contrast. Their explanations included foraminal stenosis (*n* = 7; 22.6%), disk herniation (*n* = 5; 16.1%), facet arthropathy (*n* = 5; 16.1%), disk bulging (*n* = 2; 6.5%), granulation/fibrotic tissue (*n* = 2; 6.5%), neuropathy (*n* = 2; 6.5%) and low lying or tethered spinal cord (*n* = 2; 6.5%). Though, MRI findings were inconclusive in 14 patients (55.3%).

Based on the success rates reported within these two studies, although they should be interpreted carefully, contrast-enhanced MRI seems still to be favored. The main reason is its accuracy in distinguishing between disk herniation and fibrotic tissue, of which the manner emerged as one of the most frequent causes of FBSS [13, 15]. However, the same does not apply to the early postoperative period, even though disk herniation is also believed to be the most frequent cause of early-stage FBSS [41]. Rohde et al. (2015) [42] examined immediate FBSS (iFBSS), which they defined as persistence or deterioration of symptoms after uncomplicated discectomy, emerging during postoperative hospital stay (range 3–7 days). They investigated multiple imaging techniques (i.e., MRI, CT, Myelography and post-myelographic CT). Audited by intraoperative findings, these imaging modalities correctly diagnosed 16 out of 22 recurrent/residual disk herniations (72.2%), two out of six hematomas (33.3%) and one of the two osseous stenoses (50.0%). Moreover, in six out of 13 patients (46.1%) suffering from battered nerve root syndrome, imaging showed regular postoperative changes, which was considered a correct diagnosis. Overall, 25 out of 43 (58.1%) cases were correctly diagnosed through imaging. Although MRI correctly diagnosed an epidural hematoma and two disk herniations, it failed to recognize one epidural hematoma, one battered nerve root syndrome and one fascicle herniation. No specifications were disclosed regarding the other imaging modalities. Even though the reported rate of 58.1% correct diagnoses by Rohde et al. (2015) in iFBSS patients might have been higher if (contrast-enhanced) MRI was practiced in all patients, they appeared to have drawn the correct conclusion, i.e., the value of neuroradiological imaging is of low benefit to assess FBSS during the early postoperative period [43, 44]. Conclusively, it may be hypothesized that early postoperative images indicating recurrent or pseudo disk herniation are not declarative or predictive regarding clinical outcomes.

## MR neurography in FBSS

**Fig. 10:**
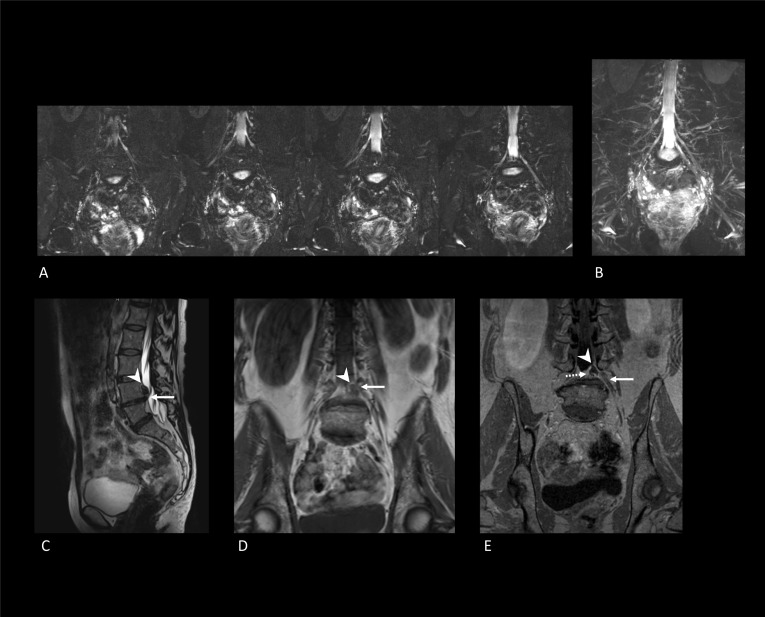
MR neurography examination of two FBBS patients. **A** Subsequent coronal 3D short tau inversion recovery sequence (3D STIR) images of one patient with no abnormalities. **B** Maximum-intensity projection of the 3D STIR images of the same patient (as depicted in **A**) showing no abnormalities of the lumbar plexus. **C** Sagittal T2-weighted MRI of a different patient showing a disk herniation occupying the left subarticular zone at level L4–L5 (white arrowhead). The white arrow depicts the coursing spinal nerve L4 on the left. **D** Coronal T1-weighted MRI of the same patient (as depicted in **C**, **E**) showing the disk herniation (white arrowhead) and left L4 spinal nerve (white arrow). **E** Coronal T1-weighted post-contrast MRI showing enhancing tissue (white dotted arrow) surrounding the disk herniation (white arrowhead). In addition, subtle perineural enhancement of the left L4 spinal nerve (white arrow) can be appreciated

FBSS often presents itself with neuropathic pain resulting from a (lumbosacral) radicular pain syndrome. Since regular MRI primarily assesses the presence of compressed nerve tissue by surrounding structures (e.g., disk herniation) and not the condition of the nerve itself, its findings are not always conclusive on whether a neuropathy is responsible for the patient’s symptoms. Instead, neuropathies are mainly identified using electrodiagnosis and clinical examination. However, magnetic resonance neurography (MRN) is a non-invasive imaging technique for dedicated assessment of the nerve’s structure instead of its function, and its refined spatial resolution and soft-tissue contrast provide an exceptional assessment of deep structures such as the muscles which form the deep internal rotators of the hip (i.e., the piriform muscle) and the lumbosacral plexus [45] (Fig. 10A, B). Hence, MRN is not only used to assess peripheral nerve entrapments and impingements but also to evaluate the localization and grading of nerve injuries and lesions. Also, the MRN’s exquisite soft-tissue contrast affords the diagnosis of non-neurological pathologies [46]. Standard MRN protocols include T1- and T2-weighted images, a 3D short tau inversion-recovery sequence, and a 3D reversed fast imaging with a steady-state precession sequence. Additionally, diffusion tensor imaging protocols and high-resolution balanced steady-state sequence images can be included. The use of intravenous gadolinium contrast agents has been described as restricted for assessing nerve infection or perineural tumor growth [47, 48] (Fig. 10).

A study among FBSS patients showed that MRN led to a substantial change in diagnosis or treatment in 12.0% and 48.0% of the FBSS cases, respectively [40]. This substantial change was defined as novel findings on MRN that changed the clinical diagnosis, and concerning treatment as “*from conservative to invasive treatment*” or “*from one type of invasive treatment to another*.” Additional findings on MRN were reported in 91% of the cases and included neuropathy (*n* = 17; mostly related to lumbosacral nerve root and sciatic nerve), hamstring tendinopathy/tear (*n* = 10), hip pathology (*n* = 8), granulation/fibrotic tissue (*n* = 6), piriformis muscle hypertrophy/atrophy (*n* = 6), foraminal or canal stenosis (*n* = 3), arachnoiditis (*n* = 3), facet arthropathy (*n* = 2), transitional anatomy (*n* = 2), dorsal ganglionopathy/injury (*n* = 2), disk herniation (*n* = 1) and tethered spinal cord (*n* = 1). These additional findings were ascribed to the superior image resolution of MRN as well as the larger field of view. Though findings resulting from the larger field of view (e.g., hamstring pathologies) may not be directly comparable to the initial MRI results, these findings were perhaps unnoticeable on MRI. One of the MRN imaging protocols with superior resolution (i.e., 0.65 mm) and 3D imaging showed relatively fewer cerebrospinal fluid pulsation artefacts, which highlighted intrathecal adhesions and the cauda equina separately. In that way, arachnoiditis and intrathecal fibrosis were more easily recognized. The additionally observed nerve injuries on MRN were detected because of related dural irregularities, pre- and postganglionic changes of nerve signal and caliber sideways to the previous surgery site.

Although symptoms related to lumbosacral plexus neuropathy are not always present, even if multiple nerves are involved, and often coexists with other pain syndromes, MRN is still able to identify underlying pathologies in most cases [49]. We reckon this could also have been the main reason for the promising results shown by Dessouky et al. (2018), as neuropathies of the lumbosacral nerve root were among the two most reported findings on MRN in addition to MRI [40]. Similar results concerning the diagnostic value of MRN were found in unilateral radiculopathies [50] and peripheral neuropathies [51]. Following this, patients suffering from radiculopathy, pelvic pain or groin pain reported significantly reduced pain intensity scores after they had received another treatment guided by findings observed on MRN [46]. Since all of these patients underwent multiple imaging examinations (MRI and CT) without a conclusive diagnosis, MRN should be considered (earlier) in patients suffering from chronic pain syndromes in order to reduce redundant imaging, as well as subsequent unsuccessful treatments and healthcare costs. The need for such thorough follow-up is further emphasized because painful sensations may alter over time [52].

MRN could also be of value in the prevention of FBSS or during surgical planning. Congenital nerve root anomalies are difficult to detect preoperatively and are considered a cause of FBSS [53], as their limited mobility makes them become damaged more easily by intraoperative manipulation [54]. Additionally, up to 60% of the FBSS cases may be caused by an inaccurately diagnosed foraminal stenosis [20]. For the diagnosis of both pathologies, DWI-MRN was reported to be superior to MRI and CT myelography. Coronal DWI-MRN images appeared to enhance accurate identification of multi-level congenital anomalies of the lumbosacral plexus and high nerve root take-off angles simultaneously [53]. As the latter could occur secondary to disk herniation, spondylolisthesis, or osteophytes, appropriate identification of the primary pain-causing pathology could result in better surgical planning.

## Emerging imaging possibilities: panacea or pie in the sky?

### Functional MRI in FBSS patients

In coincidence with the previous literature on chronic LBP patients [55], fMRI revealed that FBSS patients have an overall reduction in functional connectivity (FC) in regions that construct the default mode network (DMN) [56]. The DMN is a brain network used when the brain is at wakeful rest [57]. Contrarily, increased FC in brain regions involved in pain processing were reported, including the lateral and medial pain network [56]. Although usually absent, a functional connection between the lateral pain network and the DMN was disclosed. Similar observations were reported in chronic patient patients suffering from ankylosing spondylitis [58]. These findings suggest cross-network FC between the DMN and other brain regions involved in pain processing, which may be one of the contributors to the inducement and preservation of chronic pain, as chronic pain is also experienced in rest (i.e., in the absence of stimulus-evoked input). Another study focused on three task-positive fMRI networks, which were believed to play a role in the detection of external stimuli, cognitions and the integration of sensory and motor signals. Again, increased FC for pain-related brain regions was observed in FBSS patients, consistently across all three networks [59]. Enhanced FC in brain regions associated with sensorimotor integration (i.e., pre- and postcentral gyri) was linked to the lateral pain network, which could contribute to FBSS patients reporting increased body awareness and, thus, more carefully coordinating body movement to avoid provoking painful sensations [56]. Research in FBSS and chronic LBP patients showed that the altered FC exhibits a reversible relationship, exposed by mindfulness-based therapies [60], cognitive behavioral therapy [61], surgery [62] and SCS [63]. These outcomes suggest a causal relationship between chronic pain and abnormal fMRI findings. In conclusion, fMRI should be used to further clarify brain regions involved in the occurrence and preservation of FBSS-related symptoms. Although fMRI focuses on pain perception, whereas FBSS symptomatology is believed to arise from peripheral lesions, such objective measures could substantiate or perhaps even refine other types of diagnostics.

### Nuclear neuroimaging in FBSS patients

Nuclear neuroimaging is considered a developing field within imaging FBSS patients and comprises a variety of functional imaging techniques. To the authors’ knowledge, their use as a diagnostic tool in FBSS has never been investigated. However, SPECT of the human brain has been used to assess blood flow as a marker of neural activity. In one study, technetium-99m hexamethylpropyleneamine oxime coupled to white blood cells was injected in 18 FBSS patients to assess blood flow before and after SCS implantation. It was observed that patients who responded poorly to SCS elicited increased thalamic blood flow at baseline imaging. They also found that thalamic blood flow remained elevated after SCS treatment [64]. Another nuclear neuroimaging tool in the radiologist’s armamentarium concerns positron emission tomography (PET). PET can be carried out with different radioligands, which allows for imaging different biological processes. For example, the use of PET radiotracers which selectively label the upregulation of a peripheral benzodiazepine receptor in microglia, which occurs in neuropathic pain patients, has been proposed as a technique to visualize the state of the central nervous system and predict outcome following SCS treatment [65]. Another option concerns using the widely available fluorodeoxyglucose (FDG) for PET neuroimaging. When using FDG-PET imaging, it has been described that in patients with SCS, burst stimulation of the SCS device consistently modulated the medial pain pathway [66]. Although both SPECT and PET neuroimaging show promising results with regard to screening of FBSS patients under consideration for SCS, other studies reaffirming or contradicting these results have not followed.

### Artificial Intelligence based analysis of imaging data of FBSS patients

As identifying the source of pain is challenging in FBSS patients, imaging is often practiced, despite its low likelihood of elucidating a possible explanation. One reason for inconclusive imaging findings could be the subtle underlying pathologies that are too obscure to diagnose through the currently used imaging modalities [40]. Besides, the symptoms may arise from a combination of abnormalities that could be too challenging to be detected by solely humans [67].

A particular subcategory of the AI, convolutional neural networks (CNNs), was developed to automatically learn discriminative features from imaging data, allowing them to estimate complex nonlinear relationships without the need for feature predefinition, as well as to quantify phenotypic characteristics [68]. Moreover, CNNs assess complex data patterns in a reproducible way, resulting in lower intra- and inter-observer variability. The field of Radiomics exploits similar data quantification algorithms to explore possible relationships between patient-reported symptoms, clinical outcomes and imaging features [69]. Even those that are undetectable to the naked eye [70]. Before exploring declarative imaging features for FBSS symptomatology, CNNs that could fully characterize the lumbar region need to be developed first. Only a few studies have addressed the automatic segmentation of the lumbar region [71], including the vertebrae [72, 73], intervertebral disks [72–74], neural foramina [72] and dural sac [74]. Despite the promising accuracy-related outcomes, they primarily focused on pathological lumbar spine images. As human anatomy is highly variable, which implies a significant difficulty in the assessment and segmentation of imaging data [75], the lack of integration of nonpathological lumbar spine images lowers clinical significance. A more versatile approach, based on MR data, is needed before any of these CNNs can be utilized for unravelling explanatory imaging features in FBSS patients. Lastly, AI could also aid in monitoring any disease-related alterations if certain quantified imaging features are re-assessed over time [76].

## Conclusion

Although many imaging studies have been performed in FBSS patients, they are primarily focused on the preoperative setting or are not correlated with the patient’s symptoms in order to assess whether FBSS was indeed present and, if so, what imaging could have offered in FBSS patients specifically. Therefore, more evidence concerning imaging in FBSS patients is warranted, particularly for determining the source of pain and planning of follow-up treatment.

The current educational review outlined that spinal imaging in the postoperative setting in patients in whom FBSS might be present can be performed with conventional MR sequences. When soft tissue masses are observed in the postoperative spinal canal, post-contrast T1-weighted images can help to distinguish disk herniation from epidural fibrosis. MR neurography could be used to assess nerve structure, although its refined spatial resolution and soft tissue contrast also allow for assessing deep pelvic structures such as the muscles that form the hip’s deep internal rotators. Based on literature findings, MR neurography seems mostly suitable to find other explanations for persistent pain after spinal surgery. The clinical utility of SPECT/CT remains elusive. However, promising results have been disclosed in a small study during preoperative preparation of revisionary fusion surgery. Theoretically, imaging biomarkers seem to hold future potential in exploring relationships between imaging features and FBSS symptomatology and could be used to screen patients under consideration for SCS treatment. However, robust, well-powered prospective studies are warranted.

## Data Availability

All collected and analyzed data are included in the manuscript.

## Abbreviations

CNN: Convolutional neural network
CT: Computed tomography
DMN: Default mode network
FBSS: Failed back surgery syndrome
FC: Functional connectivity
LBP: Low back pain
MRI: Magnetic resonance imaging
MRN: Magnetic resonance neurography
SPECT: Single photon emission computed tomography
PET: Positron emission tomography
